# The American Journal of Science and Arts

**Published:** 1853-01

**Authors:** 


*The American Journal of Science and Arts.* Conducted by Professors B. Silliman, B. Silliman, Jr., and James D. Dana. Aided in the Departments of Chemistry and Physics, by Dr. Wolcott Gibbs. Second Series. No. 42. Nov., 1852. With four plates. New Haven, Conn: Published by the Editors.

It were any thing but complimentary to the intelligence of our readers to commend to their knowledge, or regard, the above periodical. It has for a long period been the chief, and indeed almost the only serial, in this country, devoted to subjects of general science; and in its scientific character we suppose it to be well understood among those most competent to judge, that it compares most favorably with similar publications in any, and all countries. It is conducted by gentlemen holding rank among the foremost of those distinguished for scientific attainments; its collaborators are their compeers in this country and abroad, and no pains are spared to render the work worthy of science and our country. Among the scientific subjects treated of in its pages, are those in which most members of the medical profession feel an interest, and respecting which information, to say the least, becomes an accomplishment very appropriate to the physician. Hence it is that a considerable share of its support, both in the way of patronage and contributions, comes from medical men. We regret to learn that it does not enjoy a circulation due to its merits, and to its character as the representative of American scientific literature. The expenses of its publication are great, and are scarcely more than repaid by the annual avails of the subscription returns. This should not be; and we take the liberty of appealing to those of our readers who do not restrict their studies to subjects exclusively medical, to subscribe for the work, if their names are not already on the subscription list.

It is a bi-monthly publication, each No. containing about 150 pages.

The subscription price is $5 *per annum.*

Orders, with remittances, may be addressed to Silliman & Dana, New Haven, Conn.; or to Phinney & Co., local agents at Buffalo.

